# Perspectives on cancer care in older patients in France

**DOI:** 10.3332/ecancer.2020.1103

**Published:** 2020-09-15

**Authors:** Mathilde Hauchecorne, Monica Pierro, Etienne Brain, Michael Bringuier, Pierre Soubeyran, Camille Chakiba, Simone Mathoulin-Pelissier, Elena Paillaud, Romain Geiss, Benjamin Besse, Loïc Mourey, Hélène Vincent, Capucine Baldini

**Affiliations:** 1 Senior Oncology Unit, Medical Oncology Department, Gustave Roussy Cancer Campus, Chevilly-Larue 94550, France; 2 Medical Oncology Department, Gustave Roussy Cancer Campus, Villejuif, France; 3 Department of Medical Oncology, Institut Curie, Saint-Cloud 92210, France; 4 Department of Medical Oncology, Institut Bergonié, Université de Bordeaux, Inserm U1218, 33076 Bordeaux Cedex, France; 5 INSERM CIC1401, Institut Bergonie, Comprehensive Cancer Center, F-33000 Bordeaux, France; 6 APHP, Paris Cancer Institute CARPEM, Department of Geriatrics, European Georges Pompidou Hospital, Paris 75015, France; 7 Univ Paris Est Creteil, INSERM, IMRB, F-94010 Creteil, France; 8 Department of Medical Oncology, Institut Claudius Regaud, Institut Universitaire du Cancer Toulouse—Oncopole, Toulouse 31059, France; 9 Drug Development Department, Gustave Roussy, Villejuif 94800, France

**Keywords:** geriatric oncology, older, geriatric assessment, cancer, aging, global health, health services, France

## Abstract

In France, cancer is the leading cause of death. Its incidence is increasing due to the growing population and longer life expectancy. Although older adults represent most new cases, they remain underrepresented in clinical trials. Their prognosis is often worse due to delayed diagnosis and multimorbidities. Geriatric oncology has made great strides worldwide, highlighted by important studies implementing geriatric assessment in clinical research and supported by the successive national cancer plans. This paper reviews the most important actions taken in France during the last decade to improve the management of older patients with cancer.

## Changes in age structure of the French population and cancer incidence

**Figure 1.**
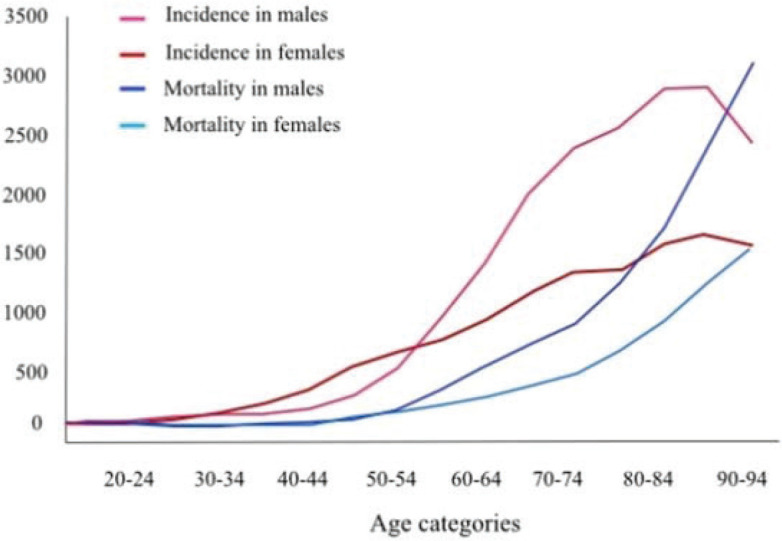
Incidence and mortality rate by age categories in France in 2018—all cancers—National Cancer Institute France.

As in the rest of the Western world, cancer incidence in France is increasing due to the growing population (+20% in 18 years) and the longer life expectancy [1] (Figure 1). Median age at diagnosis is 68 years in males and 67 years in females.

**Figure 2.**
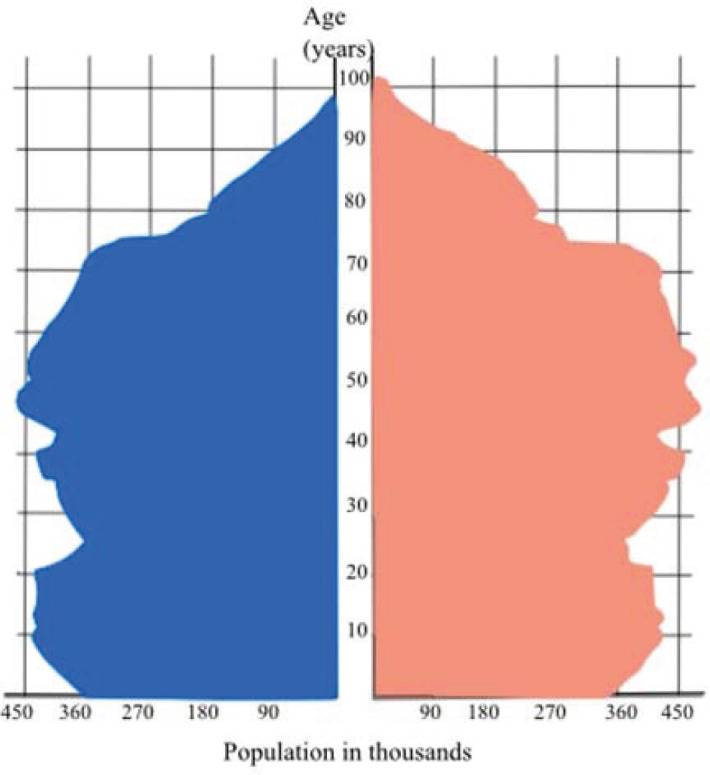
Population pyramid in France (January 2020) Insee

In January 2020, French population was estimated over 67 million, more than 20% being aged 65 or older (Figure 2) [1].

In 2017, 62% of new cancer cases were diagnosed in patients aged 65-year old or over and 11.5% in patients aged 85-year old or over [1].

Cancer prognosis in older patients is often worse than in younger ones, due to late diagnosis and multimorbidities competing for overall survival and limiting treatment [2–5].

## Geriatric oncology in France

The development of GO in France relies on the joint efforts of two professional communities, oncology and geriatrics, supported politically and financially by public authorities, including the Institut National du Cancer (INCa). It has led naturally to the creation of a scientific society and to the promotion of specific research through a specific intergroup labelled by INCa.

For the past two decades, cancer care in the older patient has been identified as a major public health issue in France. Therefore, it has been repeatedly highlighted as a priority in the successive national ‘Cancer Plans’ launched in 2003, with a 5.2 M€ annual budget dedicated to specific and targeted actions.

**Figure 3.**
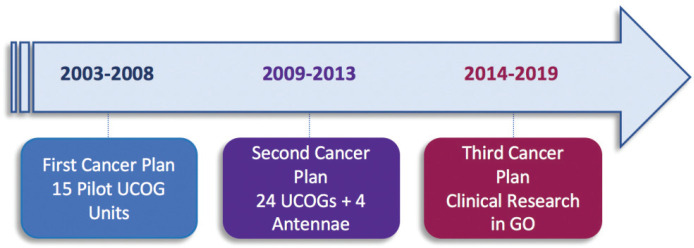
Cancer Plan timeline in France. UCOGs: geriatric oncology units; AOG: geriatric oncology offshoot units; OG: geriatric oncology.

The first ‘cancer plan’ (2003–2008), led to the creation of 15 Unités Pilotes de Coordination en Onco-Gériatrie (UPCOG, pilot units in geriatric oncology). Following a national call, this decision was supported by the INCa and the Direction Générale de l’Organisation des Soins, a branch of the French Ministry of Health. These UPCOG brought together oncologists and geriatricians in defined geographical areas, in order to spark mutual collaboration to improve the management of older patients with cancer. Training, public information and research were also listed as their missions, but the first task was to gather professionals in the same network. In most cases, these UPCOG were built on a longstanding or more recent collaboration established between one oncologist and one geriatrician, often involving the local comprehensive cancer centre and a University Hospital (Figure 3). The support to the Oncodage study to develop a screening strategy was instrumental to this first period [6].

**Figure 4.**
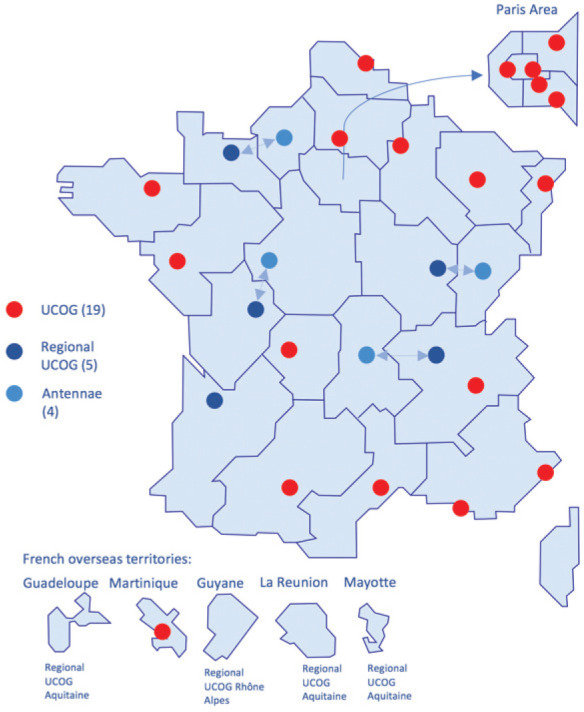
UCOGs repartition in France – National Cancer Institute.

The second cancer plan (2009–2013) confirmed the 15 nominated UPCOG as Unités de Coordination en Onco-Gériatrie (UCOG), expanding their number to 24 in order to cover the whole territory, including overseas territories. It also added four lighter antennae structures (AOG) (Figure 4). All four core and initial UPCOG missions (cares organization, training, information for the general public and research) were strengthened and further developed, aiming at spreading geographically the concept of geriatric oncology in order to establish a strong network, involving all relevant healthcare structures (Table 2). More actions were conducted in education for oncologists and geriatricians, with creation of several University Diplomas in GO and identification of specific modules in other disease-oriented diplomas (Figure 3). Results from Oncodage with the G8 screening tool contributed to set new strategies investigating the value of geriatric interventions as in the PREPARE study ongoing (NCT02704832).

The third cancer plan (2014–2019) continued to work in the same directions but stressed increasingly on the development of specific clinical research. It granted the first label of intergroup for clinical research in GO to DIALOG, joining the forces of GERICO, a long-established cooperative multidisciplinary group and the large UCOG network (Figure 3).

## The Société Francophone d’Onco-Gériatrie (SoFOG)

SoFOG is a scientific society dedicated to the management of older patients with cancer. It was launched in 2010 following 6 years of workshops Echanges Pratiques en Onco-Gériatrie on specific aspects of cancer cares in older patients, taking the opportunity of the strong political support to geriatric oncology to bring together all stakeholders.

Its main goal is to promote a multidimensional and multidisciplinary approach of older patients with cancer, based on screening for frailty and subsequent geriatric assessment reviewing areas where issues are frequent in older persons: comorbidities, polypharmacy, functional and cognitive decline, etc. This strategy allows stratifying patients in fit, vulnerable/prefrail and frail groups, depending on the level of frailty reversibility, and offering adjusted and coordinated treatment plans accordingly [7, 8].

Other actions of SoFOG include the promotion of research dedicated to the older patients with cancer, the support to education of healthcare professionals and general population, and the participation to large public debates with health authorities as INCa or Ministry of Health…).

SoFOG is also involved in the elaboration, adaptation and diffusion of guidelines in geriatric oncology, in coordination with those established by the International Society of Geriatric Oncology [9–11].

Enabling dedicated multidisciplinary meeting and access to innovative drugs are also an important mission.

## Dialogue Intergroupe pour la personnALisation de la prise en charge en OncoGériatrie (DIALOG)

In 2014, two structures decided to combine forces in a joint intergroup of clinical research in GO, the intergroup DIALOG:

- The cooperative group GERICO, founded in 2002, dedicated to clinical research in geriatric oncology and part of Unicancer. Unicancer is a hospital network entirely devoted to fighting against cancer: it includes 18 French Comprehensive Cancer Centres governed by private statutes and being non-for profit hospitals, and other hospitals.
- The large INCa-accredited UCOG network (under the auspices of the SoFOG with its Scientific Committee)

Labelled by INCa in 2014, the DIALOG intergroup has become the main actor to lay out the groundwork and foundations of such collaboration between oncology and geriatrics, in order to strongly promote specific actions in clinical research for older cancer patients at national and international levels, in response to Actions 2.16 and 5.2 of Plan Cancer 2014–2019.

Its main missions include the development of tailored designs and methodology that take into account specific expectations and unmet medical need, which cannot be adequately covered by standard developments, listing ranking research questions by priority.

It is made of three main committees, one for disease-oriented questions, one for more transversal questions, and one providing biostatistics expertise through the PACAN platform.

A first initiative led by DIALOG has been the definition of a minimal data set known as Geriatric Core Dataset to share in any trial involving older populations in order to be able to compare results [12].

It also has the mission to develop collaborations with other cooperating groups at national and international levels (Table 3).

## Conclusion

For 17 years, care for older patients with cancer has been a priority for the French healthcare authorities, as highlighted in cancer plans and calls for research grants such as PHRC, etc. Through successive supportive actions (UPCOG, UCOG, Oncodage program, GERICO program and studies, DIALOG Intergroup, etc.), this has allowed the creation of a large network covering the whole territory where specific actions of care organisations, information, education, and research can be disseminated.

It contributes to redressing inequalities of access of older patients to clinical research all across the country. Nevertheless, the number of clinical trials does not correlate with the epidemiology of cancer in older patients and effort in research and clinical knowledge needs to be continued.

## Conflicts of interest

The authors state no conflicts of interest regarding this publication.

## Funding

None.

## Figures and Tables

**Table 1.** Cancer mortality in France according to age, tumour type and sex in 2017.

	All population ³ 65-year old	All population ³ 85-year old	Men ³ 65-year old	Women ³ 65-year old
Cancer deaths (%)	115,158 (75%)	37,305 (25%)		
Cancer deaths by tumour type Lung Prostate Breast Colorectal			14,198 7,801 7,679	6,306 8,653 7,213

**Table 2.** UCOG missions.

1. The four UCOG core missions
2. Develop a better coordination for cancer cares in the older population, relying on: a strong collaboration between oncologists and geriatricians, using a screening tool for frailty as G8 as the gateway or minimum starting point to any cancer treatment decision-making implementing geriatric assessment and related information to adjust cancer treatment or add specific geriatric intervention
3. Develop research in geriatric oncology
4. Develop training in geriatric oncology (University diploma, masterclasses)
5. Disseminate information regarding cancer in older patients in the general public

**Table 3.** Studies in collaboration with other cooperating groups in France.

Trial	Setting	Tumor type	Phase	Intervention	Trial identification
**ELAN UNFIT**	first line recurrent or metastatic	head and neck squamous cell cancer	II	personalized treatment according to geriatric assessment	NCT01884623
**PRODIGE 34 - ADAGE**	Adjuvant	stage III colorectal cancer	III	personalized adjuvant chemotherapy according to geriatric assessment	NCT02355379
**ASTER70**	Adjuvant	ER+ HER2- breast cancer	III	Benefit of adjuvant chemotherapy for estrogen receptor-positive HER2-negative breast cancer in women over 70 according to genomic grade	NCT01564056
**PAMELA 70**	Metastatic	pancreatic cancer	II	efficacy and tolerance of adjusted FOLFIRINOX in elderly patients	NCT02143219
**GERICO 10**	Metastatic	castration resistant prostate cancer	II	docetaxel every 3 weeks or docetaxel weekly	NCT01254513
**NACRE GERICO 12**	Neoadjuvant	rectal cancer	III	chemoradiotherapy or short course radiotherapy	NCT02551237
